# Multiple Chronic Conditions and Provision of Services in Residential Care Communities

**DOI:** 10.1093/geroni/igaa057.299

**Published:** 2020-12-16

**Authors:** Christine Caffrey, Jessica Lendon

**Affiliations:** National Center for Health Statistics, Hyattsville, Maryland, United States

## Abstract

This study describes the relationships between the number of selected chronic conditions among residents and the number and provision methods of services provided by residential care communities (RCCs). Estimates are from the 2016 wave of the National Study of Long-Term Care Providers conducted by the National Center for Health Statistics. Chronic conditions were measured by whether the RCC reported having at least one resident with any of the most common chronic conditions: dementia, diabetes, depression, or heart disease. Services included were mental health, social work, therapeutic, dietary, and skilled nursing. Each service type was categorized by provision method (provided by employees, arrangement or referral only, or not provided). Among RCCs, 63% had all four conditions among residents, 23% had three, 12% had one to two, and 1% had none. About 66% of RCCs provided all five services, 16% provided four, 15% provided 1-3, and 3% provided none. Of the 63% of RCCs that had all four conditions among residents, 69% provided all five services, 29% provided 1-4, and 2% provided none. In these RCCs, a greater percentage provided dietary (69%) and skilled nursing services (33%) with employees compared to the other methods; a greater percentage provided therapeutic (85%) and mental health services (83%) solely through arrangement or referral compared to the other methods. This study found that, in 2016, RCCs with multiple selected conditions among their residents tended to provide a greater number of services for managing chronic conditions. How these RCCs provide services varied based on service type.

